# Prevalence of cardiovascular disease risk factors among a Nigerian adult population: relationship with income level and accessibility to CVD risks screening

**DOI:** 10.1186/s12889-015-1709-2

**Published:** 2015-04-18

**Authors:** Victor Maduabuchi Oguoma, Ezekiel Uba Nwose, Timothy Chas Skinner, Kester Awharentomah Digban, Innocent Chukwu Onyia, Ross Stuart Richards

**Affiliations:** School of Psychological and Clinical Sciences, Charles Darwin University, Darwin, Northern Territory 0909 Australia; School of Community Health, Charles Sturt University, NSW, Australia; Department of Public and Community Health, Novena University Ogume, Delta State, Nigeria; Onyx Hospital and Maternity Ltd, Lagos State, Nigeria

**Keywords:** CVD risk factors, Socio-economic status, Nigeria

## Abstract

**Background:**

In Nigeria, reports on the prevalence of modifiable cardiovascular disease (CVD) risk factors are scarce. In addition, socio-economic status (SES), an important component of the socioeconomic gradient in CVD and its risk factors has not been clearly elucidated. This study sought to assess the prevalence of CVD risk factors and how the difference in prevalence and accessibility to CVD risk screening across income levels and educational backgrounds contributes to disease diagnosis in rural and urban Nigerian adults.

**Methods:**

A cross sectional study was carried out on a sociocultural ethnic group of persons living in rural and urban settings. All participants were aged ≥ 18 years. The WHO STEPS questionnaire was used to document the demographics, history of previous medical check-up or screening, anthropometric and biochemical measurements of the participants. Average income level and educational status were indicators used to assess the impact of SES. Multivariate analyses were performed to assess any difference between the geographical locations and SES indicators, and prevalence of CVD risk factors and access to CVD risk screening.

**Results:**

The 422 participants (149 males and 273 females) had mean age (± standard deviation) of 38.3 ± 20.5 and 42.9 ± 20.7 years, respectively. Only total cholesterol (*p* = 0.001), triglyceride (*p* = 0.005), high density lipoprotein cholesterol (HDL) (*p* < 0.0001), body mass index (BMI) (*p* = 0.03) and average income rate (*p* = 0.01) showed significant difference between gender groups. Overall prevalence of prediabetes (4.9%), diabetes (5.4%), hypertension (35.7%), low HDL (17.8%), hypertriglyceridemia (23.2%), hypercholesterolemia (38.1%) and central obesity of 52.2% was recorded. Except between total cholesterol (*p* = 0.042) and HDL (*p* = 0.017), other CVD risk factors did not show a statistical significance across income levels. Participants with ‘university and postgraduate education’ had higher access to blood pressure and blood glucose screening compared to other educational groups; and this showed a statistical significance.

**Conclusion:**

This study has shown that a significant number of modifiable CVD risk factors exist in the rural and urban migrants of an adult Nigerian population. While income level did not affect the CVD risk factor prevalence, it did affect accessibility to CVD risk screening. There is a need for access to diagnosis of modifiable risk factors at all levels of society.

## Background

The prevalence of risk factors for cardiovascular disease (CVD) is on the increase in the developing nations of the world. Worldwide, CVD account for the majority of deaths due to chronic diseases [1], and more than 80% of the global burden of CVD will occur in low- and middle-income countries [2].

Socio-economic status (SES) is a predictor of CVD and its risk factors. However, the nature of this relationship varies dependent on the economic development of the countries [3-6]. In high income countries, the evidence points to an inverse relationship between SES and CVD risk factors in the adult population, regardless of indicators of SES used [7,8]. This trend differs in low-middle-income countries and among those of lower SES in the developed countries where lower SES is a potential marker of poor health outcomes [9].

In Nigeria, few reports on the association between SES and cardio-metabolic syndrome varies. High prevalence of cardiometabolic risk factors was found in high SES groups than in low SES groups [10-12], while Mbada et al. [13] noted a higher prevalence of obesity in the lower SES of a semi-urban Nigerian population. These few reports studied SES as an entity; however, the component of the SES driving the trend of the relationship in prevalence is worth exploring in relation to how it affects risk factors screening.

The objective of this study is to assess the prevalence of CVD risk factors and how the difference in prevalence and accessibility to CVD risk screening across income levels and educational backgrounds contributes to disease diagnosis in rural and urban Nigerian adults.

## Methods

### Ethical consideration

In addition to ethical approval from Human Research Ethics Committee of Novena University and the Local Government Ministry of Health at Kwale, Delta State Nigeria, clearance for this study was approved by the Human Research Ethics Committee of Charles Darwin University, Australia (HREC Reference: H14003). Prior to each day’s sample collection, a public lecture were delivered to the participants describing all information regarding the study. Information sheets and consent forms were dispatched to the participants. All participants provided written informed consent, either by signing or thumb printing on the consent form before they were enrolled for the study.

### Study design and participants

This study is a population-based cross-sectional study conducted between May and July 2014. It is part of phase one of the Prediabetes and Cardiovascular Complication Study (PACCS), which has been previously described [14,15]. One of the intents is to recruit participants to be followed up for the second and third phases of the ‘bigger’ research. The target population was adult residents from ≥ 18 years of age. Two stage cluster sampling technique was employed on a sociocultural ethnic group of persons living in rural and urban settings. For each cluster, all 18 year old students in selected secondary schools were sampled. Other participants within each cluster were enrolled through school premises, primary health care centres and town halls. Information about the study and days for screening were conveyed to the respective communities through town criers, churches, community leaders and school principals. The rural populations were drawn from Abbi (representative of remote cluster) and Kwale (representing government headquarter cluster of the rural area) communities in Ndokwa West Local Government Area (LGA) Delta State, while the urban population were indigenes of Ndokwa West LGA living in Lagos State Nigeria. Scheduled screening exercises were carried out at designated school premises, primary healthcare centres and town halls in Abbi and Kwale, Nigeria. Onyx Hospital and Maternity was the screening centre for Lagos State participants. Those meeting the inclusion criteria: ≥18 year of age, apparently healthy, verifiable contact address and residents/indigene of study area were included in the study.

### Anthropometric measurements

For assessment of obesity, waist circumference was measured midway between iliac crest and coastal margin. Height and weight were measured using a wall mounted stadiometer (Seca®, USA) and human weighing scale (Precision Hana®, India), respectively. The height and weight measurement readings were used to determine the body mass index (BMI) [16]. This was further grouped into underweight (<18.5 kg/m^2^), normal weight (18.5 – 24.9 kg/m^2^), overweight (25.0 – 29.9 kg/m^2^) and obese (>30 kg/m^2^). The waist circumference (WC) threshold for sub-Saharan ethnicity by Joint Scientific Statement on Harmonizing the Metabolic Syndrome [17] was also used to describe obesity status (Yes or No). In males, WC >94 cm was classified as obesity status ‘Yes’ while in females WC >80 cm is obesity status Yes. For hypertension, systolic and diastolic blood pressure readings were taken three times with a digital blood pressure machine (Omron®, Australia). The first blood pressure reading was discarded, taking average of the second and third readings. Guideline established by the International Diabetes Federation (IDF) [17,18] was used to define hypertension in this study groups, because recent consensus statement on harmonizing the metabolic syndrome recommended the cut-points used in the study for sub-Saharan ethnicity corresponds to the IDF criteria [3]. Hypertension was classified as systolic blood pressure reading ≥130 mmHg and/or diastolic blood pressure of ≥85 mmHg.

### Blood tests

All participants were instructed to fast overnight for at least 8 hours prior to collection of the fasting blood sample. Those who reported fasting below the given timeline were grouped to be tested using random blood sample as described by the American Diabetes Association [19]. The cut-offs were 100 - 125 mg/dL (prediabetes) and ≥126 mg/dL (diabetes) for impaired fasting glucose. 140 – 199 mg/dL (prediabetes) and ≥200 mg/dL for random blood sample. Classification of the lipid profile parameters (Total Cholesterol, triglyceride and high density lipoprotein cholesterol) followed the IDF criteria [18]. Boundary values ≥200 mg/dL indicates hypercholesterolemia, ≥150 mg/dL (hypertriglyceridemia), ≤40 mg/dL (low HDL in men) and ≤50 mg/dL (low HDL in women). Specimens for biochemical analysis were fresh capillary whole blood collected by finger prick. CardioChek® Professional Analyser was used to measure blood glucose level and lipid profile according to manufacturer’s instructions.

### Demographics and other questionnaire-based variables

The WHO STEPS questionnaire for non-communicable diseases surveillance was adopted to document the demographics, behavioural characteristics (tobacco and alcohol use, diet, level of physical activity and history of diseases), anthropometric and biochemical measurements of the participants [20]. Demographic characteristics, including gender, age in years, highest level of education completed, main work status, average income earnings and number of people >18 years in same household were self-reported and recorded on the questionnaire during a face-to-face interview. Average income earnings reported in Nigerian Naira, but were converted to the International US dollar using the Google Finance currency converter database on 23rd October 2014 (₦1 = US$0.0061) and categorized into five groups: low income (<$109/month), low-mid income ($110 – $310/month); upper-mid income ($311 – $640/month) and high income (>$645/month).

### History of previous medical check-up or screening

Participant’s accessibility to CVD risk screening was reported during the face-to-face interview. Participants were asked whether or not they have ever had their blood pressure, blood sugar, and cholesterol measured by a doctor or other health workers. Dichotomous no/yes responses were recorded in the questionnaire.

### Statistical analysis

Sample size was calculated using the StatCalc application from the Epi-Info software (version 7.1, CDC Atlanta USA). Assuming 25% response distribution at 5% confidence limits (margin of error), the study sample size would be approximately 203 at 90% confidence level or 68 at 90% confidence level for each cluster. These were below our actual study sample size. Frequency table for the demographic information were determined. Prevalence of CVD risk factors and history of CVD risk screening were compared between gender and stratified age groups. Chi-square test was used to test for significance.

Multivariate analyses were performed in two phases. First, dichotomous ‘yes/no’ response variables including history of blood sugar, blood pressure, and lipid profile check-ups were analysed to find if there exist any difference between the various groups (educational status, income level and location). Secondly, continuous variables including BMI, WC, diastolic and systolic blood pressure, as well as blood glucose (BG), high density lipoprotein cholesterol (HDL), total cholesterol (CHOL) and triglycerides (TG) were analysed. Each of the two phases of data analyses were performed thrice to evaluate the relationship with three factors, which included:

1. Income status categorized in 4-groups – low income (<$109/month), low-mid income ($110 – $310/month); upper-mid income ($311 – $640/month) and high income (>$645/month). Excluded ‘unknown group’ were majorly participants who have no form of employment/income and those who declined to indicate.
2. educational status categorized in 4-groups - ‘no formal school’ and ‘less than primary education’ were grouped together to form a group, ‘university’ and ‘postgraduate education’ were also regrouped together, while primary and secondary education stood alone.
3. geographical location categorized into 3-groups (urban and two rural communities).

Fisher’s Least Significant Difference (LSD) post hoc analysis was employed to determine mean difference of subgroups of educational status, income level and geographical location with CVD risk factors and history of blood sugar, blood pressure and lipid profile check-ups. Level of significance was set at 0.05.

## Results

### Baseline characteristics of participants

Study participants included 149 men and 273 women. The distribution and characteristics of demographic variables among the three cluster groups are summarized in Tables 1 and 2. In all the three study locations, the number of female participants was higher than the male counterpart. The mean age amongst participant was 42.9 ± 20.7 and 38.3 ± 20.5 for females and males, respectively, with females accounting for 64.7% of the total participants. Participants in the urban location (Lagos) had the highest average daily income of 21.33 ± 4.758 US$. There was a statistically significant difference between gender subgroups in terms of average daily income (p < 0.05). Abbi community (rural) recorded 29.7% of participants without any form of formal education and this was the highest among the three studied geographical locations. The urban participants were more educated and received higher incomes than the rural counterparts. Among gender, total cholesterol (*p* = 0.001), triglyceride (*p* = 0.005), HDL (*p* < 0.0001), BMI (*p* = 0.03) and average income rate (*p* = 0.01) showed statistical significant difference.

**Table 1:** **Demographic information**

**Characteristics**	**Abbi [** ***n*** **(%)]**	**Kwale [** ***n*** **(%)]**	**Lagos [** ***n*** **(%)]**
***Gender***			
Male	56 (38.6)	51 (28.2)	42 (43.8)
Female	89 (61.4)	130 (71.8)	54 (56.3)
Total	145 (34.4)	181 (42.9)	96 (22.7)
***Age group***			
18-24	34 (23.8)	106 (58.6)	11 (11.7)
25-34	10 (7.0)	21 (11.6)	8 (8.5)
35-44	17 (11.9)	19 (10.5)	25 (26.6)
45-54	15 (10.5)	27 (14.9)	27 (28.7)
55-64	11 (7.7)	8 (4.4)	17 (18.1)
65-74	17 (11.9)	0 (0.0)	4 (4.3)
≥75	39 (27.3)	0 (0.0)	2 (2.1)
*Missing variables*	*2*	*0*	*2*
***Education completed***			
No formal school	43 (29.7)	6 (3.4)	1 (1.0)
Less than primary school	4 (2.8)	2 (1.1)	4 (4.2)
Primary school	68 (46.9)	95 (53.1)	35 (36.5)
Secondary school	19 (13.1)	46 (25.7)	36 (37.5)
University	10 (6.9)	24 (13.4)	17 (17.7)
Postgraduate	0 (0.0)	6 (3.4)	3 (3.1)
Refused	1 (0.7)	0 (0)	0 (0.0)
*Missing variables*	*0*	*2*	*0*
***Work status***			
Government employee	7 (4.8)	28 (15.6)	2 (2.1)
Non-government employee	3 (2.1)	1 (0.6)	17 (17.7)
Self-employed	23 (15.9)	24 (13.3)	58 (60.4)
Non-paid	58 (40.0)	14 (7.8)	0 (0.0)
Student	31 (21.4)	106 (58.9)	7 (7.3)
Homemaker	2 (1.4)	0 (0.0)	2 (2.1)
Retired	6 (4.1)	0 (0.0)	5 (5.2)
Unemployed (able to work)	6 (4.1)	6 (3.3)	4 (4.2)
Unemployed (unable to work)	8 (5.5)	1 (0.6)	1 (1.0)
Refused	1 (0.7)	0 (0.0)	0 (0.0)
*Missing variables*	*0*	*1*	*0*
***Average daily income (US$)***			
Low income	41 (28.3)	21 (11.6)	20 (20.8)
Low-middle income	15 (10.3)	15 (8.3)	25 (26.0)
Upper-middle income	3 (2.1)	5 (2.8)	10 (10.4)
High income	1 (0.7)	4 (2.2)	14 (14.6)
Unknown	85 (58.6)	136 (75.1)	27 (28.1)

**Table 2:** **Average (Mean ± SD) levels of demographic, anthropometric and biochemical parameters**

	Cluster subgroups*			**Gender subgroups**	
	**Abbi**	**Kwale**	**Lagos**	**Female**	**Male**
**Age (years)** ^**†**^	51.27 ± 2.03	28.24 ± 1.0	45.1 ± 13.4	42.9 ± 20.7	38.3 ± 20.5
**Average daily income** ^**†**^	3.79 ± 0.61	8.80 ± 1.58	21.33 ± 4.758	19.47 ± 4.78	7.39 ± 1.33
**WC (cm)**	87.31 ± 0.93	83.11 ± 0.99	94.1 ± 16.3	86.7 ± 11.5	87.2 ± 15.1
**CHOL(mg/dL)** ^**†**^	208.16 ± 8.70	191.05 ± 6.52	173.0 ± 16.3	174.4 ± 76.6	202.0 ± 82.5
**G (mg/dL)** ^**†**^	137.42 ± 5.54	120.71 ± 4.18	117.5 ± 56.2	136.1 ± 61.6	118.5 ± 53.2
**HDL (mg/dL)** ^**†**^	73.04 ± 2.29	75.29 ± 1.86	63.4 ± 20.4	64.4 ± 24.5	75.8 ± 22.5
**BMI (kg/m** ^**2**^ **)** ^**†**^	23.41 ± 0.43	24.05 ± 0.49	25.2 ± 5.0	23.3 ± 4.6	24.5 ± 6.3
**Systolic (mmHg)**	120.94 ± 2.25	119.20 ± 2.76	133 ± 19.8	124.0 ± 21.9	122.4 ± 34.7
**Diastolic (mmHg)**	72.76 ± 2.44	69.93 ± 0.90	80.0 ± 12.4	72.7 ± 12.7	73.5 ± 23.1
**FBG (mg/dL)**	101.76 ± 5.99	80.95 ± 2.12	88.2 ± 26.6	95.6 ± 48.1	88.3 ± 45.8
**RBG (mg/dL)**	116 ± 0.0	92.34 ± 2.92	103.4 ± 29.6	102.9 ± 29.7	94.8 ± 26.4

Significant level α = 0.05; FBG = Fasting blood glucose, RBG = Random blood glucose, CHOL = total cholesterol, TG = Triglycerides, WC = waist circumference, HDL = high density lipoprotein cholesterol, BMI = body mass index.

*Statistical significance levels between subgroups presented in Table 4.

^†^statistically significant difference between gender subgroups.

All missing data variables were excluded from the analysis.

### Prevalence of CVD risk factors and CVD risk screening/check-ups across gender and age-groups

**Figure 1:**
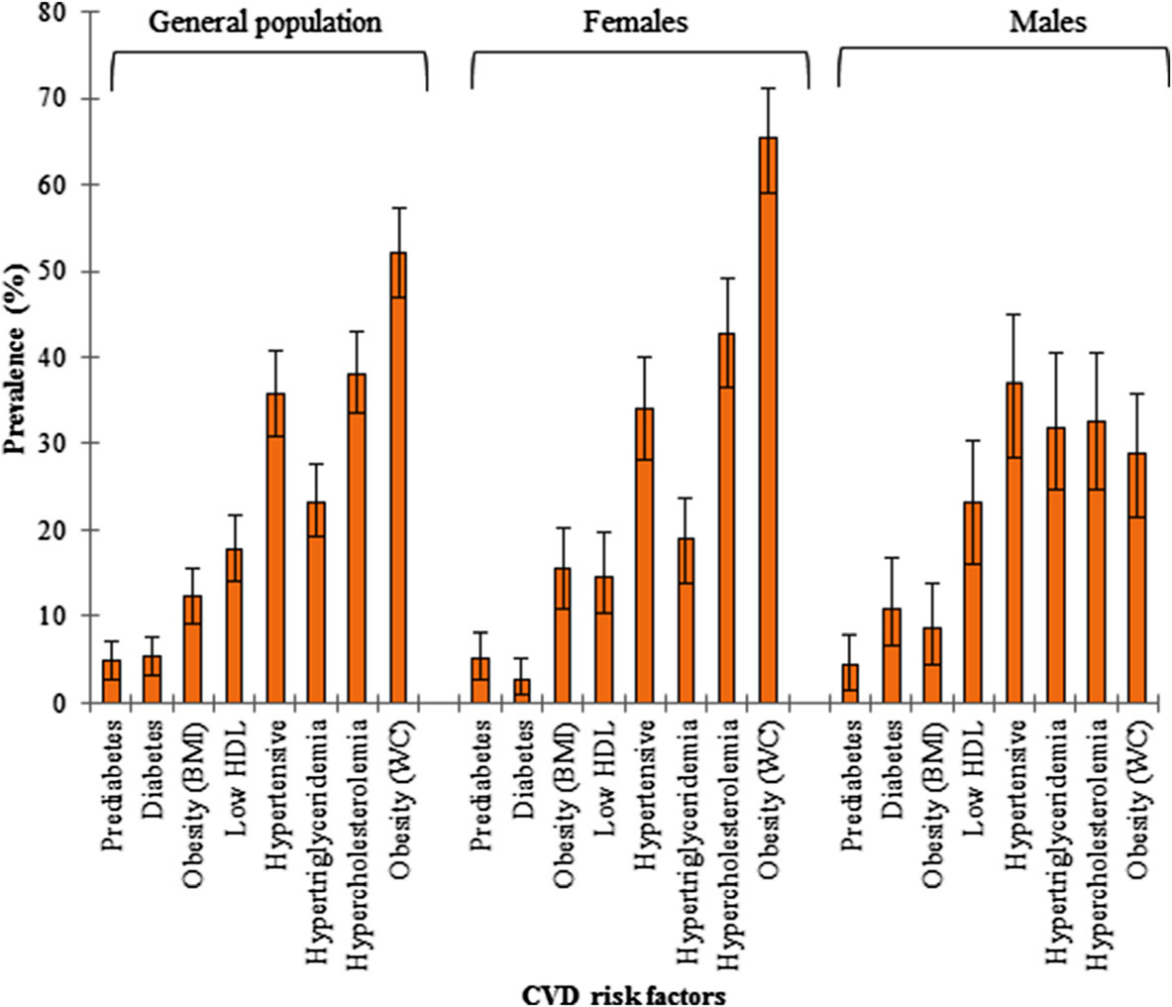
Prevalence of CVD risk factors across gender and general population.

**Figure 2:**
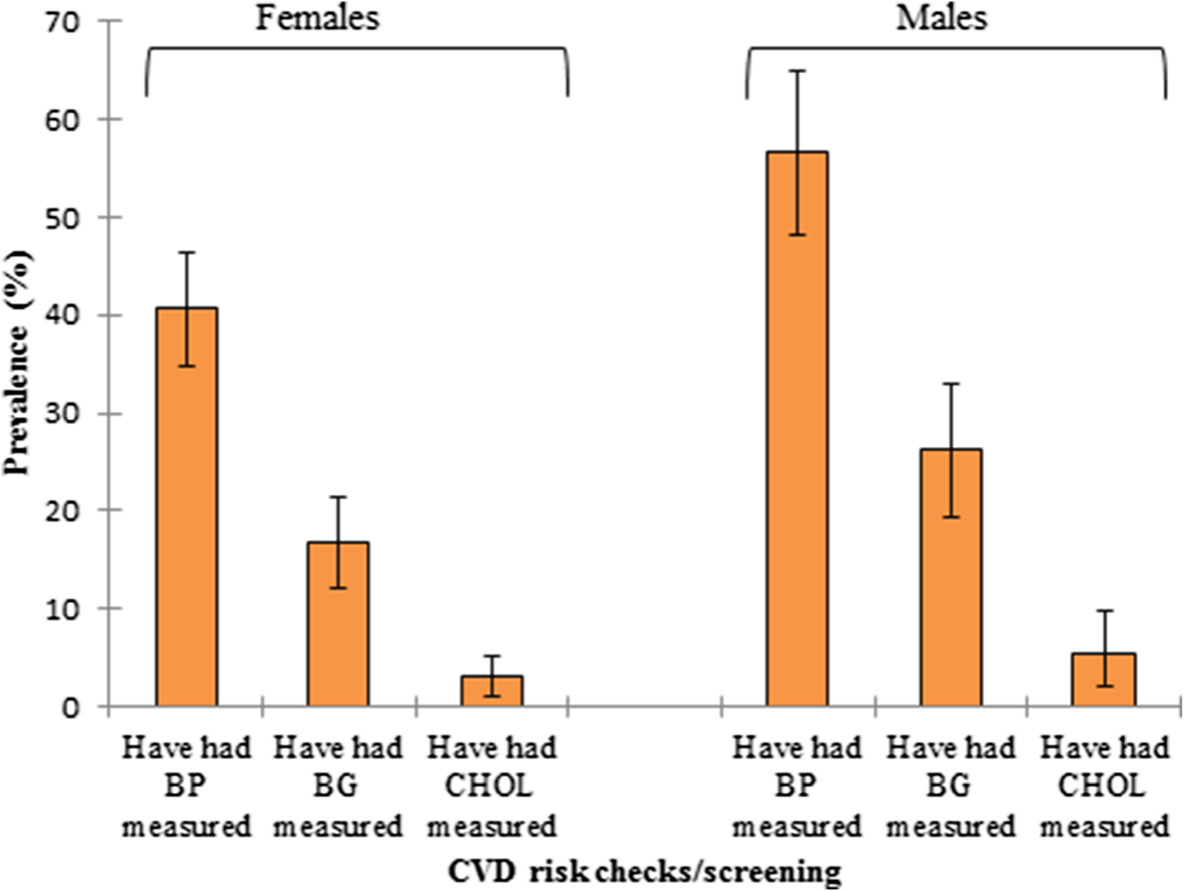
Percentage of participants who have had CVD risk checks/screening among gender.

Prevalence of prediabetes in the entire study population was 4.9% (95% CI 2.7-7.0%) based on the cut-off standards adopted in the study. Overall prevalence of diabetes in our study is 5.4% (95% CI: 3.2-7.6%). Among females, prevalence of prediabetes and diabetes was 5.2% (95% CI: 2.6 – 8.2%) and 2.6% (95% CI: 0.9 – 5.2%), respectively. Prevalence of diabetes was higher in males (10.9%; 95% CI: 6.5 – 16.7%) than in females (p = 0.003). Also, the results show that obesity (based on BMI and/or WC cut-off points) appears to be more prevalent in females than males; while hypertension and dyslipidaemia were more in males than females (Figure 1). More males have had their blood pressure, blood glucose and cholesterol levels checked than females, but statistical significant difference only exist between males and females in blood pressure (*X*^*2*^; *p* = 0.002) and blood sugar checks (*X*^*2*^; *p* = 0.028) (Figure 2).

Further, the lower age groups 18 – 24 and 25 – 34 years had the highest prevalence of low HDL of 19.7% (95% CI: 13.2-27.0%) and 34.3% (95% CI: 20.0-51.4%), respectively. A sizeable prevalence of prediabetes was found to exist at the lower age groups, with the highest prevalence seen to occur at 34–45 years old participants. The prevalence of hypertension and central obesity increased with increase in age, peaking at age group 54 – 64 years (Table 3A). Across all age groups, the mostly accessed CVD risk screening was blood pressure checks (*X*^*2*^; *p* < 0.0001) while the least accessed was cholesterol (lipid profile) checks (*X*^*2*^; *p* = 0.094) (Table 3B).

**Table 3:** **Prevalence of CVD risk factors and accessibility to CVD risk screening across age groups**

**A. CVD risk factors**	**Age group in years (95% confidence interval)**							**p-values**
	**18-24**	**25-34**	**35-44**	**45-54**	**55-64**	**65-74**	**≥75**	
**↑TG**	10.2 (5.1-15.6)	11.4 (2.9-20.0)	22.4 (10.2-32.7)	32.3 (21.0-43.5)	46.9 (28.1-63.8)	37.5 (16.2-62.5)	48.4 (29.0-67.7)	<0.0001
**↑CHOL**	23.4 (16.1-30.7)	22.9 (11.4-35.5)	32.7 (21.6-46.9)	48.4 (33.9-61.3)	59.4 (40.6-75.0)	62.5 (37.5-81.3)	74.2 (56.7-90.3)	<0.0001
**Obesity (BMI)**	2.2 (0.0-4.4)	17.1 (5.7-28.6)	16.3 (6.1-28.6)	32.3 (21.0-43.5)	15.6 (6.3-28.1)	12.5 (0.0-31.3)	12.9 (3.2-25.8)	<0.0001
**↓HDL**	19.7 (13.1-27.0)	34.3 (20.0-51.4)	20.4 (10.2-34.7)	14.5 (6.5-24.2)	12.5 (3.1-25.0)	6.3 (0.0-25.0)	6.5 (0.0-17.5)	0.053
**Prediabetes**	3.6 (0.7-7.3)	2.9 (0.0-11.4)	8.2 (2.0-16.3)	6.5 (1.6-12.9)	0.0	0.0	6.5 (0.0-16.1)	0.398
**Diabetes**	0.7 (0.0-2.9)	0.0	4.1 (0.0-10.2)	8.1 (1.6-14.5)	18.8 (6.3-31.3)	25.0 (6.3-50.0)	6.5 (0.0-16.1)	<0.0001
**Hypertension**	7.3 (2.9-11.7)	28.6 (14.3-42.9)	40.8 (26.5-55.1)	54.8 (38.7-66.1)	68.8 (50.0-84.4)	56.3 (37.5-81.3)	71.0 (54.8-85.2)	<0.0001
**Obesity (WC)**	16.8 (10.2-22.6)	51.4 (34.3-68.6)	77.6 (64.5-88.6)	79.0 (67.7-88.7)	87.5 (75.0-96.9)	68.8 (43.8-87.5)	64.5 (48.4-80.6)	<0.0001
**B. CVD risk screening**								
**Have had BP measured**	6.7 (3.4-11.4)	43.6 (28.2-61.5)	70.5 (59.0-82.0)	86.4 (78.8-93.9)	83.3 (69.4-94.4)	65.0 (45.0-85.0)	51.2 (36.6-65.9)	<0.0001
**Have had BG measured**	3.4 (0.7-6.7)	7.7 (0.0-15.4)	26.2 (14.8-37.7)	36.4 (24.2-47.0)	55.6 (38.9-69.4)	35.0 (15.0-55.0)	19.5 (7.3-31.7)	<0.0001
**Have had CHOL measured**	1.3 (0.0-3.4)	0.0	4.9 (0.0-11.5)	4.5 (0.0-10.6)	11.1 (2.8-22.2)	5.0 (0.0-15.0)	7.3 (0.0-17.1)	0.094

BP = blood pressure, BG = blood glucose, CHOL = cholesterol.

TG = triglycerides, CHOL = total cholesterol, BMI = body mass index, HDL = high density lipoprotein cholesterol, WC = waist circumference, ↑ = hyper, ↓ = low.

### Prevalence of CVD risk factors and CVD risk screening/check-ups across subgroups of geographical location, average daily income and level of education

Prevalence of hypercholesterolemia was higher in the two rural populations 51.8% (95% CI: 41.4-61.3) and 34.8% (95% CI 27.4-42.9) than the urban area 29.3% (95% CI: 19.7-40.2), while the urban had higher prevalence of central and overall obesity and hypertension. Overall, the highest prevalence of prediabetes, diabetes, low HDL and hypertriglyceridemia was recorded in Abbi community, which is the most remote of the study locations. Apart from blood glucose, other assessed CVD risk variables showed significant difference between the urban population and rural populations. Also, a statistical significance exists between the rural subjects and the urban in terms of having had access to CVD risk screening (Tables 4 and 5).

**Table 4:** **Percentage prevalence of CVD risk factors and accessibility of CVD risks screening across subgroups of geographical location, average daily income and level of education among study population**

**A: CVD risk factors**		**↑TG (95% CI)**	**↑CHOL (95% CI)**	**Obese (BMI) (95% CI)**	**↓HDL (95% CI)**	**Prediabetes (95% CI)**	**Diabetes (95% CI)**	**Hypertension (95% CI)**	**Obese (WC) (95% CI)**
**Geographical location**	**Abbi**	32.7 (22.7-42.2)	51.8 (41.4-61.3)	8.2 (2.7-12.7)	19.1 (12.7-26.4)	6.4 (2.3-10.9)	10.9 (5.5-16.4)	37.3 (28.2-47.3)	51.8 (41.8-61.8)
	**Kwale**	19.5 (13.8-25.9)	34.8 (27.4-42.9)	14.0 (9.1-19.2)	17.1 (11.0-23.2)	3.7 (1.2-6.7)	1.8 (0.0-4.3)	23.2 (16.8-29.9)	40.2 (32.3-48.2)
	**Lagos**	19.6 (12.0-28.7)	29.3 (19.7-40.2)	17.4 (9.8-26.1)	17.4 (9.8-26.1)	4.3 (1.1-8.7)	5.4 (1.1-9.8)	53.3 (43.0-64.1)	72.8 (63.0-81.5)
**Average daily income (US$)**	**Low income**	28.4 (18.9-39.2)	45.9 (33.8-58.1)	17.6 (8.9-27.0)	14.9 (8.1-23.0)	8.1 (2.7-14.9)	4.1 (0.0-8.1)	54.1 (41.9-66.2)	81.1 971.6-89.8)
	**Low-middle income**	29.8 (17.0-44.7)	44.7 (31.9-57.4)	21.3 (10.6-34.0)	19.1 (8.5-31.9)	2.1 (0.0-6.4)	6.4 (0.0-14.9)	46.8 (31.9-59.6)	66.0 (51.1-78.7)
	**Upper-middle income**	17.6 (0.0-35.3)	35.3 (11.8-52.9)	11.8 (0.0-29.4)	11.8 (0.0-29.4)	11.8 (0.0-29.4)	0.0	52.9 (29.4-76.5)	47.1 (23.5-67.1)
	**High income**	44.4 (19.9-66.7)	38.9 (16.7-61.1)	16.7 (0.0-33.3)	33.3 (11.1-55.6)	0.0	22.2 (5.6-38.9)	33.3 (11.1-55.6)	83.3 (64.4-100)
**Education completed**	**No formal education and Less than primary education**	40.8 (26.5-55.9)	69.4 (57.1-82.5)	10.2 (2.0-20.4)	12.2 (4.1-21.3)	2.0 (0.0-6.1)	6.1 (0.0-14.3)	59.2 (46.9-73.5)	71.4 (57.1-83.7)
	**Primary education**	16.0 (11.2-21.7)	29.7 (22.6-36.6)	10.3 (6.3-15.4)	16.6 (10.9-22.3)	4.0 (1.7-7.1)	4.6 (1.7-8.2)	24.6 (17.5-31.4)	41.1 (34.3-48.6)
	**Secondary education**	19.3 (10.9-27.3)	31.8 (22.3-42.0)	12.5 (5.7-19.3)	21.6 (12.5-31.2)	6.8 (2.3-12.5)	6.8 (2.3-12.5)	34.1 (23.9-44.3)	53.4 (43.2-63.6)
	**University and Postgraduate**	39.6 (26.4-52.8)	50.9 (37.7-63.0)	24.5 (13.2-35.8)	18.9 (8.7-30.2)	5.7 (0.0-13.2)	5.7 (0.0-12.1)	47.2 (33.2-60.4)	66.0 (52.8-79.2)
**B: History of CVD risk screening**				**Ever had BP measured?**		**Ever had BG measured?**		**Ever had CHOL measured?**	
				**Yes** **(95% CI)**	**No (95% CI)**	**Yes (95% CI)**	**No (95% CI)**	**Yes (95% CI)**	**No (95% CI)**
**Geographical location**		**Abbi**		45.5 (37.5-53.1)	54.2 (46.9-62.5)	18.9 (12.6-24.8)	81.1 (75.2-87.4)	3.5 (0.7-6.3)	96.5 (93.7-99.3)
		**Kwale**		32.4 (25.3-39.2)	67.6 (60.8-74.7)	11.9 (7.4-17.0)	88.1 (83.0-92.6)	1.7 (0.0-3.6)	98.3 (96.4-100)
		**Lagos**		72.9 (64.6-81.3)	27.1 (18.8-35.4)	36.5 (27.1-44.8)	63.5 (55.2-72.9)	8.3 (3.1-14.6)	91.7 (85.4-96.9)
**Average daily income (US$)**		**Low income**		61.3 (50.0-70.5)	38.8 (29.5-50.0)	27.5 (18.8-37.5)	72.5 (62.5-81.3)	5.0 (1.3-10.0)	95.0 (90.0-100)
		**Low-middle income**		72.7 (60.0-83.6)	27.3 (16.4-40.0)	30.9 (18.2-42.6)	69.1 (57.4-81.8)	9.1 (1.8-18.2)	90.9 (81.8-98.2)
		**Upper-middle income**		83.3 (66.7-100)	16.7 (0.0-33.3)	33.3 (16.7-55.6)	66.7 (44.4-83.3)	5.6 (0.0-16.7)	94.4 (83.3-100)
		**High income**		100.0	0.0	57.9 (34.7-78.9)	42.1 (21.1-65.2)	5.3 (0.0-15.8)	94.7 (84.2-100)
**Education completed**		**No formal education and Less than primary education**		48.3 (35.5-62.1)	51.7 (37.9-64.5)	20.7 (10.3-31.0)	79.3 (69.0-89.7)	5.2 (0.0-12.1)	94.8 (87.9-100)
		**Primary education**		30.1 (23.5-36.7)	69.9 (63.3-76.5)	13.8 (8.7-18.4)	86.2 (81.6-91.3)	1.5 (0.0-3.6)	98.5 (96.4-100)
		**Secondary education**		56.4 (45.5-66.3)	43.6 (33.7-54.5)	22.8 (14.9-32.7)	77.2 (67.3-85.1)	5.0 (1.0-9.9)	95.0 (90.1-99.0)
		**University and Postgraduate**		79.7 (69.5-89.8)	20.3 (10.2-30.5)	35.6 (23.7-48.2)	64.4 (51.8-76.3)	8.5 (1.7-16.0)	91.5 (84.0-98.3)

BP = blood pressure, BG = blood glucose, CHOL = cholesterol.

TG = triglycerides, CHOL = total cholesterol, BMI = body mass index, HDL = high density lipoprotein cholesterol, PD = prediabetes, DB = diabetes, HYP = hypertension, WC = waist circumference, ↑ = hyper, ↓ = low.

**Table 5:** **Fisher’s Least Significant Difference (LSD) post hoc multivariate analysis within subgroups of geographical location, average daily income and education: in association with CVD risk factors and history of disease screening**

**CVD risk factors**	**Independent variables**		**Mean difference**	**Standard error**	**p-value**	**95% confidence interval**	
						**Lower bound**	**Upper bound**
**Waist circumference**	**Abbi**	**Kwale**	**4.02** ^*^	1.675	.017	.73	7.31
		**Lagos**	**−6.65** ^*^	1.919	.001	−10.42	−2.87
	**Kwale**	**Lagos**	**−10.67** ^*^	1.765	.000	−14.14	−7.20
**Total cholesterol**	**Abbi**	**Kwale**	**20.86** ^*^	9.963	.037	1.27	40.46
		**Lagos**	**37.89** ^*^	11.415	.001	15.44	60.34
	**Kwale**	**Lagos**	17.02	10.496	.106	−3.62	37.66
**Triglycerides**	**Abbi**	**Kwale**	**18.39** ^*^	6.981	.009	4.66	32.12
		**Lagos**	**20.56** ^*^	7.999	.011	4.83	36.28
	**Kwale**	**Lagos**	2.17	7.355	.768	−12.30	16.63
**HDL cholesterol**	**Abbi**	**Kwale**	−1.34	2.890	.644	−7.02	4.35
		**Lagos**	**9.55** ^*^	3.311	.004	3.03	16.06
	**Kwale**	**Lagos**	**10.88** ^*^	3.044	.000	4.90	16.87
**Body mass index**	**Abbi**	**Kwale**	−0.80	0.705	.260	−2.18	0.59
		**Lagos**	**−1.91** ^*^	0.808	.019	−3.50	−0.32
	**Kwale**	**Lagos**	−1.11	0.743	.135	−2.57	0.35
**Systolic blood pressure**	**Abbi**	**Kwale**	0.72	3.763	.849	−6.69	8.12
		**Lagos**	**−11.45** ^*^	4.312	.008	−19.93	−2.97
	**Kwale**	**Lagos**	**−12.17** ^*^	3.965	.002	−19.97	−4.37
**Diastolic blood pressure**	**Abbi**	**Kwale**	2.88	2.481	.246	−1.99	7.76
		**Lagos**	**−6.32** ^*^	2.843	.027	−11.91	−0.73
	**Kwale**	**Lagos**	**−9.20** ^*^	2.614	.000	−14.34	−4.06
**Blood glucose**	**Abbi**	**Kwale**	**16.12** ^*^	5.088	.002	6.12	26.13
		**Lagos**	6.76	5.830	.247	−4.71	18.22
	**Kwale**	**Lagos**	−9.37	5.360	.081	−19.91	1.17
**Waist circumference**	**Low income**	**Low-mid income**	1.20	2.479	.629	−3.68	6.08
		**Upper-mid income**	1.51	3.575	.673	−5.52	8.54
		**High income**	−3.61	3.493	.302	−10.48	3.26
	**Low-mid income**	**Upper-mid income**	0.31	3.762	.935	−7.09	7.71
		**High income**	−4.81	3.684	.193	−12.05	2.44
	**Upper-mid income**	**High income**	−5.12	4.495	.256	−13.96	3.72
**Total cholesterol**	**Low income**	**Low-mid income**	**31.05** ^*^	15.248	.042	1.07	61.04
		**Upper-mid income**	28.06	21.987	.203	−15.18	71.30
		**High income**	22.20	21.485	.302	−20.05	64.45
	**Low-mid income**	**Upper-mid income**	−2.99	23.137	.897	−48.49	42.51
		**High income**	−8.85	22.660	.696	−53.41	35.71
	**Upper-mid income**	**High income**	−5.86	27.648	.832	−60.23	48.51
**Triglycerides**	**Low income**	**Low-mid income**	4.78	10.628	.653	−16.12	25.68
		**Upper-mid income**	9.49	15.325	.536	−20.65	39.63
		**High income**	−11.45	14.974	.445	−40.89	18.00
	**Low-mid income**	**Upper-mid income**	4.71	16.126	.770	−27.00	36.42
		**High income**	−16.23	15.794	.305	−47.28	14.83
	**Upper-mid income**	**High income**	−20.94	19.270	.278	−58.83	16.96
**HDL cholesterol**	**Low income**	**Low-mid income**	6.44	4.429	.147	−2.27	15.15
		**Upper-mid income**	1.56	6.387	.807	−11.00	14.12
		**High income**	**14.92** ^*^	6.241	.017	2.64	27.19
	**Low-mid income**	**Upper-mid income**	−4.88	6.721	.468	−18.10	8.34
		**High income**	8.48	6.582	.199	−4.47	21.42
	**Upper-mid income**	**High income**	13.36	8.031	.097	−2.44	29.15
**Body mass index**	**Low income**	**Low-mid income**	0.05	1.035	.963	−1.99	2.08
		**Upper-mid income**	1.00	1.493	.505	−1.94	3.93
		**High income**	−0.23	1.459	.874	−3.10	2.64
	**Low-mid income**	**Upper-mid income**	0.95	1.571	.546	−2.14	4.04
		**High income**	−0.28	1.539	.856	−3.31	2.75
	**Upper-mid income**	**High income**	−1.23	1.878	.513	−4.92	2.46
**Systolic blood pressure**	**Low income**	**Low-mid income**	8.47	5.609	.132	−2.56	19.50
		**Upper-mid income**	8.99	8.088	.267	−6.92	24.89
		**High income**	10.97	7.903	.166	−4.57	26.51
	**Low-mid income**	**Upper-mid income**	0.51	8.511	.952	−16.22	17.25
		**High income**	2.50	8.336	.765	−13.89	18.89
	**Upper-mid income**	**High income**	1.99	10.170	.845	−18.01	21.99
**Diastolic blood pressure**	**Low income**	**Low-mid income**	−0.99	3.783	.795	−8.43	6.45
		**Upper-mid income**	−3.42	5.455	.531	−14.14	7.31
		**High income**	−3.39	5.330	.526	−13.87	7.10
	**Low-mid income**	**Upper-mid income**	−2.43	5.740	.672	−13.72	8.86
		**High income**	−2.40	5.621	.670	−13.45	8.66
	**Upper-mid income**	**High income**	0.03	6.859	.996	−13.46	13.52
**Blood glucose**	**Low income**	**Low-mid income**	−10.24	7.782	.189	−25.54	5.06
		**Upper-mid income**	4.63	11.221	.680	−17.44	26.70
		**High income**	−21.25	10.965	.053	−42.81	.32
	**Low-mid income**	**Upper-mid income**	14.87	11.808	.209	−8.35	38.09
		**High income**	−11.00	11.565	.342	−33.75	11.74
	**Upper-mid income**	**High income**	−25.88	14.110	.067	−53.62	1.87
**Waist circumference**	**NLE**	**PE**	**6.08** ^*^	2.207	.006	1.74	10.42
		**SE**	2.90	2.437	.235	−1.90	7.69
		**UPE**	**−5.69** ^*^	2.698	.035	−11.00	−0.39
	**PE**	**SE**	−3.18	1.784	.075	−6.69	0.33
		**UPE**	**−11.77** ^*^	2.126	.000	−15.95	−7.59
	**SE**	**UPE**	**−8.59** ^*^	2.364	.000	−13.24	−3.94
**Total cholesterol**	**NLE**	**PE**	**59.68** ^*^	12.767	.000	34.57	84.78
		**SE**	**60.55** ^*^	14.097	.000	32.83	88.28
		**UPE**	17.28	15.604	.269	−13.40	47.97
	**PE**	**SE**	.87	10.316	.933	−19.41	21.16
		**UPE**	**−42.40** ^*^	12.295	.001	−66.58	−18.22
	**SE**	**UPE**	**−43.27** ^*^	13.672	.002	−70.16	−16.39
**Triglycerides**	**NLE**	**PE**	**33.61** ^*^	8.961	.000	15.99	51.24
		**SE**	**22.14** ^*^	9.895	.026	2.68	41.60
		**UPE**	−5.11	10.953	.641	−26.64	16.43
	**PE**	**SE**	−11.47	7.241	.114	−25.71	2.77
		**UPE**	**−38.72** ^*^	8.630	.000	−55.69	−21.75
	**SE**	**UPE**	**−27.25** ^*^	9.596	.005	−46.12	−8.38
**HDL cholesterol**	**NLE**	**PE**	**8.07** ^*^	3.832	.036	.53	15.61
		**SE**	**11.42** ^*^	4.231	.007	3.10	19.74
		**UPE**	8.07	4.684	.086	−1.14	17.28
	**PE**	**SE**	3.35	3.096	.280	−2.74	9.44
		**UPE**	.00	3.691	.999	−7.26	7.25
	**SE**	**UPE**	−3.35	4.104	.414	−11.42	4.72
**Body mass index**	**NLE**	**PE**	0.17	0.895	.851	−1.59	1.93
		**SE**	−1.68	0.988	.090	−3.62	.2633
		**UPE**	**−4.49** ^*^	1.093	.000	−6.64	−2.34
	**PE**	**SE**	**−1.85** ^*^	0.723	.011	−3.27	−0.43
		**UPE**	**−4.66** ^*^	0.862	.000	−6.35	−2.96
	**SE**	**UPE**	**−2.81** ^*^	0.958	.004	−4.69	−0.92
**Systolic blood pressure**	**NLE**	**PE**	**14.96** ^*^	4.949	.003	5.22	24.69
		**SE**	9.57	5.465	.081	−1.18	20.31
		**UPE**	7.06	6.049	.244	−4.84	18.95
	**PE**	**SE**	−5.39	3.999	.179	−13.25	2.47
		**UPE**	−7.90	4.766	.098	−17.27	1.47
	**SE**	**UPE**	−2.51	5.300	.636	−12.93	7.91
**Diastolic blood pressure**	**NLE**	**PE**	3.15	3.281	.338	−3.31	9.60
		**SE**	−1.42	3.623	.696	−8.54	5.71
		**UPE**	−5.05	4.010	.209	−12.93	2.83
	**PE**	**SE**	−4.57	2.651	.086	−9.78	0.65
		**UPE**	**−8.19** ^*^	3.160	.010	−14.41	−1.98
	**SE**	**UPE**	−3.63	3.514	.302	−10.54	3.28
**Blood glucose**	**NLE**	**PE**	−3.35	6.782	.621	−16.69	9.98
		**SE**	−4.07	7.489	.587	−18.80	10.65
		**UPE**	−3.19	8.289	.701	−19.49	13.11
	**PE**	**SE**	−0.72	5.480	.895	−11.50	10.06
		**UPE**	0.16	6.531	.980	−12.68	13.01
	**SE**	**UPE**	0.88	7.263	.903	−13.40	15.17
**History of CVD risk screening**	**Independent variables**		**Mean difference**	**Standard error**	**p-value**	**95% confidence interval**	
						**Lower bound**	**Upper bound**
**Ever had BP measured?**	**Abbi**	**Kwale**	−0.13*	0.054	0.017	−0.23	−0.02
		**Lagos**	0.27*	0.063	<0.0001	0.15	0.40
	**Kwale**	**Lagos**	0.40*	0.060	<0.0001	0.28	0.52
**Ever had BG measured?**	**Abbi**	**Kwale**	−0.07	0.044	0.119	−0.16	0.02
		**Lagos**	0.18*	0.052	0.001	0.07	0.28
	**Kwale**	**Lagos**	0.24*	0.050	<0.0001	0.15	0.34
**Ever had CHOL measured?**	**Abbi**	**Kwale**	−0.02	0.022	0.410	−0.06	0.02
		**Lagos**	0.05	0.025	0.057	0.00	0.10
	**Kwale**	**Lagos**	0.07*	0.024	0.007	0.02	0.11
**Ever had BP measured?**	**Low income**	**Low-mid income**	0.11	0.078	0.143	−0.04	0.27
		**Upper-mid income**	0.22	0.117	0.059	−0.01	0.45
		**High income**	0.39*	0.114	0.001	0.16	0.61
	**Low-mid income**	**Upper-mid income**	0.11	0.121	0.383	−0.13	0.34
		**High income**	0.27*	0.119	0.022	0.04	0.51
	**Upper-mid income**	**High income**	0.17	0.147	0.258	−0.12	0.46
**Ever had BG measured?**	**Low income**	**Low-mid income**	0.03	0.067	0.613	−0.10	0.17
		**Upper-mid income**	0.06	0.100	0.561	−0.14	0.26
		**High income**	0.30*	0.098	0.002	0.11	0.50
	**Low-mid income**	**Upper-mid income**	0.02	0.104	0.816	−0.18	0.23
		**High income**	0.27*	0.102	0.009	0.07	0.47
	**Upper-mid income**	**High income**	0.25	0.126	0.053	0.00	0.49
**Ever had CHOL measured?**	**Low income**	**Low-mid income**	0.04	0.034	0.225	−0.03	0.11
		**Upper-mid income**	0.01	0.050	0.912	−0.09	0.10
		**High income**	0.00	0.049	0.957	−0.09	0.10
	**Low-mid income**	**Upper-mid income**	−0.04	0.052	0.499	−0.14	0.07
		**High income**	−0.04	0.051	0.455	−0.14	0.06
	**Upper-mid income**	**High income**	0.00	0.063	0.963	−0.13	0.12
**Ever had BP measured?**	**NLE**	**PE**	−0.18*	0.070	0.011	−0.32	−0.04
		**SE**	0.08	0.077	0.292	−0.07	0.23
		**UPE**	0.31*	0.087	<0.0001	0.14	0.48
	**PE**	**SE**	0.26*	0.058	<0.0001	0.15	0.37
		**UPE**	0.49*	0.070	<0.0001	0.36	0.63
	**SE**	**UPE**	0.23*	0.077	0.003	0.08	0.38
**Ever had BG measured?**	**NLE**	**PE**	−0.07	0.059	0.248	−0.18	0.05
		**SE**	0.02	0.065	0.750	−0.11	0.15
		**UPE**	0.15*	0.073	0.042	0.01	0.29
	**PE**	**SE**	0.09	0.049	0.067	−0.01	0.18
		**UPE**	0.22*	0.059	<0.0001	0.10	0.33
	**SE**	**UPE**	0.13*	0.065	0.049	0.00	0.26
**Ever had CHOL measured?**	**NLE**	**PE**	−0.04	0.029	0.207	−0.09	0.02
		**SE**	0.00	0.032	0.944	−0.06	0.06
		**UPE**	0.03	0.036	0.354	−0.04	0.10
	**PE**	**SE**	0.03	0.024	0.149	−0.01	0.08
		**UPE**	0.07*	0.029	0.016	0.01	0.13
	**SE**	**UPE**	0.04	0.032	0.264	−0.03	0.10

TG = triglycerides, CHOL = total cholesterol, BMI = body mass index, HDL = high density lipoprotein cholesterol, PD = prediabetes, DB = diabetes, HYP = hypertension, WC = waist circumference.

*The mean difference is significant at 0.05 level, NLE = No formal education and Less than primary education, PE = Primary education, SE = secondary education, UPE = University and Postgraduate.

Highest prevalence of central obesity, hypertriglyceridemia, diabetes and low HDL was found in the high income group. Except between ‘low-middle income and low income’ groups for CHOL (*p* = 0.042), and ‘high income and low income’ groups for HDL-C (*p* = 0.017), other CVD risk factors did not show a statistical significance across income levels. Among individuals who have had access to blood pressure and blood glucose screening, statistical significance only exists between the high income group and “low-middle income and low income” groups.

There was a significant difference between individuals with ‘university and post graduate education’ and ‘no formal education and less than primary education’ and those with primary education and secondary education, respectively, in regards to prevalence of hypertriglyceridemia and hypercholesterolemia. Significant difference also exists between the ‘university and postgraduate education’ group and other groups in prevalence of obesity. Having had access to blood pressure and blood glucose screening was significantly different between the ‘university and postgraduate education’ and other educational groups (Tables 4 and 5).

## Discussion

The results from this study indicate that the adult Nigeria population bear a substantial burden of modifiable CVD risk factors. In 1960s, diabetes was considered to be rare among Nigerians and reported prevalence rates were <1% [21,22]. Few studies in different geopolitical zones of Nigeria identified sizeable prevalence rates of diabetes and prediabetes in study patients. The first reported prediabetes condition in Nigeria was in 1998 [23]. They found a prevalence of 2.2% in a group of urban adults in Nigeria. In another study carried out in an urban Southern Western Nigeria, the incidence of prediabetes in the whole study participants was 3.3% as compared to confirmed diabetic prevalence of 4.7% [24]. Another study in a rural Nigerian community identified 4.8% prevalence of diabetes [25]. There is indication that increased urban migration and urbanization over time encouraging lifestyle changes is contributory to increase in prevalence of these modifiable risk factors. It is also envisaged that with increase in reporting, the true prevalence of prediabetes and diabetes in Nigeria will be unravelled, especially in apparently healthy subjects in the rural communities.

More females were obese than males measured either by assessing the overall obesity or central obesity. This agrees with the findings of Ogunmola et al. [25] and Adegoke et al. [26], in rural Nigerian communities. In contrast to diabetes and prediabetes, the urban participants were more obese than the rural population. Early data in Nigeria during the middle and later part of last century suggested low prevalence of obesity [27,28]. The reverse is the case in present time, where more areas are becoming urbanized encouraging a sedentary lifestyle and unhealthy eating. Subjects in rural communities have farming and trading as the primary occupation, which provides substantial physical activity. This is contributory to their low prevalence of obesity as compared to the urban migrant counterparts.

Our study also found that the age group 18–24 years had a sizeable prevalence of prediabetes, hypercholesterolemia, central obesity and low HDL. In a ten-year incidence of risk of cardiovascular disease study, it was found that the increased risk in individuals with impaired fasting glucose was largely driven by the coexistence of multiple CVD risk factors [29]. This becomes alarming for a setting where algorithms for early screening and detection of disease risk factors are under assessed. It is argued that the effect of glucose lowering drugs can delay conversion of prediabetes to diabetes [30], but this can only be the case in societies with effective health systems, where individuals have adequate health awareness and health seeking behaviour that will enhance the opportunities for detection and intervention.

Higher prevalence’s of hypertension have been reported elsewhere in Nigeria [25,31,32]. Our finding on gender difference in prevalence of hypertension is also corroborated by other studies done in Nigeria [31,33]. Another study in South-eastern Nigeria supports this finding where they noted a high prevalence of hypertension and obesity among the middle age groups [34]. The strong association between obesity and hypertension highlights the central role of endothelial dysfunction, which is contributory to the initiation and progression of atherosclerosis [35,36]. It points to the importance of early diagnosis and intervention against prediabetes, hypertension and associated CVD risks and complications, especially in low-middle-income countries.

A higher percentage of males have had their blood pressure, blood glucose and cholesterol checked than females. The reason for this occurrence was not clearly known. Ahaneku et al. [31] found that more females in their study have had their blood pressure checked than males. Females more often participate in health screening exercises than males as observed in both our rural and urban populations. This trend has been reported in several studies in Nigeria [25,26,31,33]. This could be explained by the characteristics of the traditional African society where males are the major bread winners for their entire family and live in the cities while their wives and children live in the village [37].

Socio-economic factors across the study population show that rural populations are more disadvantaged in terms of high income earnings and post primary education. A greater percentage of participants in the rural setting are living in poverty, which the WHO defined in absolute terms are low income less than US$2 per day. The minimum wage in Nigeria is ₦18,000 per month, which is approximately US$109.80 per month. However, income status was not associated with high prevalence of hypertension and dyslipidaemia (triglycerides, total cholesterol and HDL) in our study population. The high income group were more diabetic and obese; these differences were not statistically significant from the lower and middle income groups. Some studies carried out in the western countries showed that individuals with lower income were more likely to be obese and diabetic [38,39]. Our finding is corroborated with a study carried out in south-western Chinese rural adults which found no evidence to support any association between income level and CVD risk factor prevalence [7].

Again, this study shows that educational level is not a protective factor against CVD risk factors. The trend shows that individuals with no primary education and those with at least a university education had higher prevalence of CVD risk factors than those with primary and secondary education. Studies elsewhere which found a low prevalence of CVD risk factors in individuals with higher educational achievement, suggests that this may be due to the fact that increased knowledge enables an individual to make healthy choices regarding dietary habits and physical activity [7,40]. Factors which may have contributed to the inability of higher educational attainment to shield against CVD risk factors in our study relates well to the fragile health systems embattling this part of the world. Inadequate health awareness programmes and public health education campaigns across all levels is a contributory factor. It is imperative to lobby companies, educational institutions, government and non-governmental organisation to engage in periodic health education programmes to enhance staffs’ health awareness and seeking behaviour.

Across the study geographical locations, more individuals in the urban setting have had their blood pressure measurements checked, than those in the rural settings. This highlights the consequences of health disparity, whereby the more affluent in society have better access to health care [41]. Income level did not affect accessibility to blood pressure checks indicating that the cost of having a blood pressure measurement in our study settings is quite affordable to all income levels. However, in all, blood sugar and cholesterol were the least accessed. Inability to have a blood sugar and cholesterol checks was not associated with rural or urban setting, income level and level of education. In our preliminary study, we found that about 64.7% of study participants do not have a clinic or hospital doctor they visit for health checks [15], and this impacts on the health literacy irrespective of the level of education attained. We also noted that participants cite various reasons for not seeking health check [15], including cost. Although this study statistically shows that income level did not influence people’s inability to access blood sugar and cholesterol checks, it does in the sense that people have formed a perpetual habit where they do not often seek health checks when they are apparently healthy couple with the fact that the cost of a lipid profile test equals the minimum wage per month. Therefore, attitudinal change which impacts positively on health outcome remains one of the public health messages that cannot be avoided.

### Limitations

The poor health seeking behaviour and nonchalant attitude amongst the study population is an impediment to covering a more robust sample size. This was further impacted upon by the limited time allocated for sample collection. The belief of not seeking health checks when not ill or when apparently healthy was one of the issues identified during the preliminary survey that is impacting negatively on the effectiveness of chronic disease management in this population [15]. Although our sample population was far above the minimum estimated sample size, it was not in par with the WHO STEPS recommendation where the minimum estimated sample size is expected to be multiplied by 2 (for gender) and by 5 (for each 10 year interval). The main interest in this reported sub-study is the association of income level and accessibility to CVD risks screening on prevalence of the risk factors of CVD. We acknowledge the high percentage of the young adults as a potential limitation in future analyses where age would be considered. Indication of our knowledge that ‘age is a factor in financial ability to access medical checkups because students and unemployed young adults would hardly be able to afford’.

## Conclusion

This study has shown that a significant prevalence of modifiable CVD risk factors exist in the rural and urban migrants of an adult Nigerian population. The early unset of these CVD risk factors among the youngest adult population clearly underlines the need for early screening and interventions. Since the level of education attained did not change the severity in prevalence rate of the risk factors, improved public health awareness across all educational levels will play a pivotal role in improving the health literacy levels in and around this population group. While income level did not affect the CVD risk factor prevalence, it did affect accessibility to CVD risk screening. There is a need for access to diagnosis of modifiable risk factors at all levels of society.
