# Erratum To: Prevalence of cardiovascular disease risk factors among a Nigerian adult population: relationship with income level and accessibility to CVD risks screening

**DOI:** 10.1186/s12889-015-2101-y

**Published:** 2015-09-14

**Authors:** Victor Maduabuchi Oguoma, Ezekiel Uba Nwose, Timothy Chas Skinner, Kester Awharentomah Digban, Innocent Chukwu Onyia, Ross Stuart Richards

**Affiliations:** School of Psychological and Clinical Sciences, Charles Darwin University, Darwin, 0909, Northern Territory Australia; School of Community Health, Charles Sturt University, NSW, Australia; Department of Public and Community Health, Novena University Ogume, Delta State, Nigeria; Onyx Hospital and Maternity Ltd, Lagos State, Nigeria

## Main body text

Unfortunately, the original version of this article [1] contained an error. The gender subgroup category of table 2 is displayed incorrectly. The category labelled ‘Female’ should be Male and the other labelled ‘Male’ should be Female.

The results subsection of the Abstract also contains a mistake as a result of the mislabel. The first sentence “The 422 participants (149 males and 273 females) had mean age (± standard deviation) of 38.3 ± 20.5 and 42.9 ± 20.7 years, respectively” should read “The 422 participants (273 females and 149 males) had mean age (± standard deviation) of 38.3 ± 20.5 and 42.9 ± 20.7 years, respectively”. This error has been corrected in the original article.

The ‘Baseline Characteristics of Participants’ subsection in the results contains an error. Reading from line 4, the sentence “The mean age amongst participant was 42.9 ± 20.7 and 38.3 ± 20.5 for females and males, respectively” should be corrected to “The mean age amongst participant was 42.9 ± 20.7 and 38.3 ± 20.5 for males and females, respectively”. We regret these errors which has now been corrected.
